# Iron is an important influence of volcanic ash input on the evolution of deep-sea ecosystems

**DOI:** 10.1128/spectrum.00715-25

**Published:** 2025-08-01

**Authors:** Shijie Bai, Zijia Wang, Yuang Guo, Hengchao Xu, Jiwei Li, Xiaotong Peng

**Affiliations:** 1 Institute of Deep-sea Science and Engineering, Chinese Academy of Sciences 383875 , Sanya, China; 2 University of Chinese Academy of Sciences, Beijing, China; Luonnonvarakeskus, Oulu, Finland

**Keywords:** volcanic ash, iron, trench, functional genes, microbial communities

## Abstract

Volcanoes, originating from deep-seated magmatic activity, serve as crucial conduits connecting Earth’s interior and surface. Volcanic eruptions, a primary manifestation of this connection, exert profound influence across Earth’s surface systems. Volcanic ash, a significant product of these eruptions, has long been a focal point of Earth science research due to its far-reaching impacts. However, the challenges associated with deep-sea sampling and a relative lack of interdisciplinary collaboration have hindered our understanding of how volcanic ash deposition affects the abyssal environment. Here, leveraging a combined geological and microbiological approach, we investigated sediments within the Kermadec Trench impacted by rhyolitic volcanic ash. Our findings demonstrate that iron availability is the primary driver of microbial community structure in these ash-influenced deep-sea sediments. The mantel test analysis further revealed that four key categories of iron-related functional genes involved in iron acquisition (heme transport, iron transport, and siderophore transport) and iron storage significantly shape the resident microbial communities. Furthermore, metagenomic binning yielded numerous refined metagenome-assembled genomes (MAGs) from these deep-sea sediments, all of which harbored iron-related functional genes. Viral metagenomic analysis suggests that viruses in these sediments do not directly influence abyssal prokaryote-mediated iron cycling through the carriage of iron-related auxiliary genes. Instead, viral lysis of iron-cycling prokaryotes appears to be a key regulatory mechanism. These results provide critical new data and insights into microbial iron cycling in the deep sea under the influence of volcanic ash deposition.

**IMPORTANCE:**

Volcanic eruptions emit vast amounts of ash, which eventually settle in the deep ocean. This study explores how the deposition of volcanic ash influences deep-sea microbial communities, primarily through iron enrichment. Our findings highlight the pivotal role of iron-related genes in shaping these communities, while viruses may play an indirect role in modulating iron cycling. These insights enhance our understanding of how volcanic activity affects deep-sea ecosystems and biogeochemical cycles. By elucidating the intricate link between volcanic ash, iron availability, and microbial dynamics, this research provides a novel perspective on how geological processes drive life in the deep ocean. Ultimately, this knowledge contributes to a deeper understanding of global nutrient cycles.

## INTRODUCTION

Volcanic eruptions produce lava flows, pyroclastic flows, and gases. Among these, pyroclastic flows, high-density mixtures of hot gases and solid pyroclastic material, represent one of the most destructive volcanic hazards, posing significant threats to both environment and climate. Fine ash (less than 2 mm) and aerosols generated by large explosive eruptions can reach the stratosphere, potentially traveling thousands of kilometers before settling and causing far-reaching regional and even global environmental impacts (1). While volcanic ash deposition in the marine environment is a complex process, settling velocities are rapid, exceeding 2 cm/s (2–4). This rapid vertical flux minimizes the residence time of volcanic ash in the water column, limiting the influence of ocean currents on its dispersal and promoting its sequential incorporation into seafloor sediments (1, 3, 5).

Volcanic ash, composed of fine particles (<2 mm), is widely dispersed across ocean basins following explosive eruptions (1, 6). Due to its high settling velocity—often exceeding 1,600 m per day—volcanic ash can reach the abyssal seafloor within days, where it becomes incorporated into marine sediments (2). This rapid and direct pathway enables volcanic ash to serve as an important source of nutrients and trace metals, including iron, phosphorus, and manganese, for deep-sea ecosystems (7). Upon entering seawater, ash-bound labile substances—such as metal salts and protonated ions—dissolve quickly, elevating nutrient concentrations (8, 9). Reactions with volcanic gases like SO₂ and HF can form soluble salt films (e.g., sulfides and halides), further enhancing nutrient release (9, 10). Experimental studies demonstrate that volcanic ash can stimulate microbial growth by releasing bioavailable elements, although shifts in microbial community structure may include reduced diversity and altered successional patterns (11–14).

However, the influence of deposited volcanic ash on deep-sea sediment microbial communities remains poorly understood. This knowledge gap likely stems from the challenges associated with deep-sea sediment collection and the need for interdisciplinary collaboration across marine geology, geochemistry, and microbiology to address this complex issue.

Our previous work in the Yap Trench demonstrated that volcanic ash layers enriched in iron-bearing minerals significantly altered sediment microbial communities and enhanced microbial iron acquisition and metal metabolism, promoting deep-sea metal cycling (15). While our previous study established these general effects of volcanic ash, it is important to note that volcanic ash is not a uniform material. Different categories of volcanic ash, upon deposition, can influence and alter sedimentary microbial community composition, structure, and function. This raises several key questions: Do deep-sea sediment microorganisms respond differently to these varying ash categories? Are there corresponding differences in community structure? Moreover, even within the same ash category, are there similarities and differences in the effects on sediment microbial communities? If differences exist, what are the primary drivers?

The Kermadec Trench, situated in a relatively oligotrophic region of the southwest Pacific Ocean, reaches a maximum depth of 10,047 m and comprises 3.6% of New Zealand’s Exclusive Economic Zone. The hadal zone of the trench (below 6,000 m) encompasses an area of 147,935 km² (16, 17). Volcanic ash from eruptions within the Taupo Volcanic Zone can be transported by winds toward the Kermadec Arc. Subsequently, these ash particles are carried northward across the Plain and eventually deposited within the Kermadec Trench, contributing to the volcanic ash sediment deposits examined in this study (18). The trench’s unique funnel-like morphology promotes sediment and organic matter accumulation, as well as sediment transport driven by gravity and seismic activity. These deposits create environments conducive to extensive microbial-mediated biogeochemical cycling (16). Here, we present mineralogical, geochemical, and microbiological analyses of surface sediment samples from the Kermadec Trench, characterizing one volcanic ash type to investigate whether microbial communities in sediments impacted by this ash type differ, and to identify the primary driving factors.

## MATERIALS AND METHODS

### Geological background and sample collection

**Fig 1:**
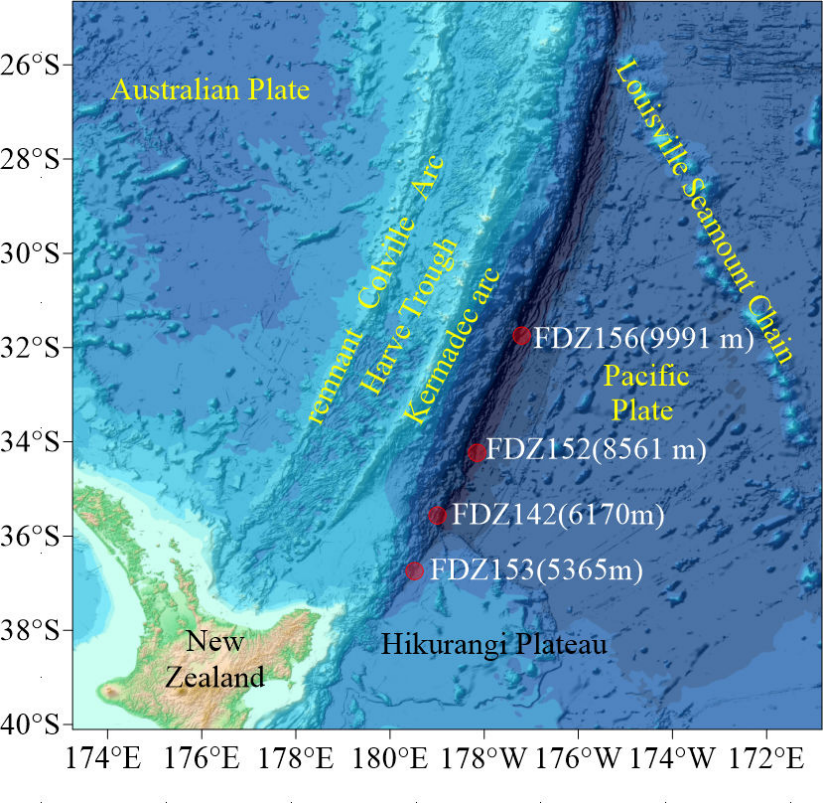
Sampling stations along the axis of the Kermadec Trench.

The Kermadec Trench, situated in the southwestern Pacific Ocean, formed by the subduction of the Pacific Plate beneath the Australian Plate, is approximately 1,195 km long, 120 km wide, and 10,000 m deep, characterized by steep adjacent slopes. Extensive flow deposits, localized volcanism, and sedimentary basins create a complex habitat for benthic fauna (16). Connected to the Hikurangi Trough and Rapuhia Scarp, the trench receives an influx of cold Antarctic water, the Lower Circumpolar Deep Water mass (LCDW), making it one of the world’s coldest trenches, with bottom temperatures ranging from 1.2°C to 1.8°C (19). Sediment samples were collected along the Kermadec Trench axis during a 2022 China-New Zealand joint cruise aboard the R/V *Tansuoyihao* using the manned submersible *Fendouzhe* (20). Sampling site information is detailed in Table 1 and Fig. 1. Surface sediments (0 to 2 cm) from four dives were designated 142_L0, 152_L0, 153_L0, and 156_L0, corresponding to the respective dive names. Pushcore samples were sectioned at 2 cm intervals onboard and stored at −20°C. One subsample was allocated for mineralogical analysis and another for microbial analysis. Following the cruise, samples were transported to the laboratory on dry ice and stored at −80°C until further processing.

**TABLE 1:** Sampling site information and relative abundances of pyroxene and hematite observed in surface sediment samples from the Kermadec Trench^*a*^

Site	Latitude	Longitude	Water depth	Augite	Hematite
	(°S)	(°W)	(m)	Particle/g-dry sediments	
FDZ156	31.754	177.1942	9991	250.2	63.5
FDZ152	34.22761	178.1519	8561	n.d	n.d.
FDZ142	35.56913	179.0033	6170	n.d	n.d.
FDZ153	36.74287	179.4729	5365	n.d	n.d.

^*a*^ Mineral abundances were estimated by visual counting under a stereomicroscope, with three replicate counts conducted for each station. “n.d.” indicates that the mineral was not detected in any of the replicates.

### Mineralogical analyses

#### Mineralogy of bulk sediments

Malvern Panalytical Empyrean X-ray diffractometer was used to obtain bulk mineralogical XRD profiles at IDSSE. The effective analytical conditions for identifying samples in the present study were as follows: Cu-Kα radiation at 40 kV and 30 mA, a curved graphite secondary monochromator, a scan from 5 to 80° (2θ), a step size of 0.01313° (2θ), and a step time of 0.5°/min.

#### Tephra separation and Raman identification

Volcanic glass, augite, and hematite particle concentrations were measured by microscopic counting and normalized to shards per gram of dry sediment (shards/g) (Table 1). Individual glass shards from each sample were hand-picked and embedded in epoxy resin, which was then sectioned and polished for Raman analysis.

Confocal Raman spectroscopy was used to support the characterization of these glasses in the local selected area. Raman analyses were performed on polished thin sections using a LabRAM HR Evolution (Horiba Jobin Yvon) Confocal Raman spectrometer equipped with a 532 nm excitation laser at IDSSE. A 600 lines/mm grating was used to obtain spectra with a spectral resolution around 4 cm^−1^. The laser intensity used is 0.2 mW, and dwell time ranges from five to 20 s. All Raman peak positions were read directly from the measured average spectra, calculated from representative regions with high signal-to-noise ratio and after background removal.

### Molecular analyses

#### Microbial DNA extraction and sequencing

DNA was extracted from four sediment samples using the DNeasy PowerSoil Pro Kit (250) (QIAGEN, USA; REF. 47016) following the manufacturer’s protocol. Extracted DNA was quantified using a Qubit fluorometer (Invitrogen, Life Technologies Holdings Pte Ltd., Singapore). The V4 hypervariable region of the 16S rRNA gene was amplified using the modified primer pair 515 f (5′GTGYCAGCMGCCGCGGTAA-3′) and 806 r (5′GGACTACNVGGGTWTCTAAT-3′) (21). PCR cycling conditions were as follows: 95°C for 3 min; 27 cycles of 95°C for 30 s, 55°C for 30 s, and 72°C for 45 s; and a final extension at 72°C for 10 min. Triplicate PCR reactions were pooled and purified using a TaKaRa purification kit (TaKaRa, Japan). Libraries were prepared using the TruSeq DNA sample preparation kit (Illumina, San Diego, CA, USA) following the manufacturer’s protocol and sequenced at MajorBio Co. Ltd. (Shanghai, China) on the Illumina HiSeq platform (Illumina) with 250 bp paired-end reads. For metagenomic sequencing, DNA was fragmented by sonication to 350 bp and ligated with Illumina sequencing adapters. Metagenomic libraries were constructed using the NEBNext Ultra DNA Library Prep Kit for Illumina (NEB, USA) following the manufacturer’s protocol. Metagenomic samples were sequenced on the Illumina HiSeq platform at Novogene (Beijing, China) to generate paired-end reads. All samples were sequenced using the Illumina NovaSeq platform (PE150), generating 150 bp paired-end reads, with 20 G of raw data per sample.

#### Amplicon and metagenomic analysis

After sequencing and obtaining the raw amplicon data, barcodes as well as forward and reverse primers (one mismatch each was allowed) were removed to obtain clean data. Paired-end reads with at least 30 bp overlap were assembled into full-length sequences using FLASH v1.2.8 (22), yielding an average fragment length of 253 bp for prokaryotes. High-quality sequences (N-free) were retained using Btrim v0.2.0 (23), selecting sequences between 245 and 260 bp for subsequent prokaryotic analysis. Amplicon sequence variants (ASVs) were generated using UNOISE3 with default settings (24). A representative sequence from each 16S rRNA gene ASV was taxonomically annotated by comparison with the SILVA 138.1 database (25), encompassing bacterial, archaeal, and eukaryotic sequences. The resulting ASV table was used for downstream analyses. To account for varying sequencing depths, ASV read counts were normalized by random resampling. Alpha diversity indices (Shannon, Inverse Simpson, Chao1 (26), and observed richness) were calculated using R v4.3.3 (27) and Mothur (28) to assess microbial community diversity in surface sediment samples from different sites. Beta diversity and community structure differences were analyzed using principal coordinate analysis (PCoA) based on unweighted and weighted UniFrac distances (29).

After obtaining the raw metagenomic sequencing data, the adapters were removed using fastp v0.23.4 software, and the data were subjected to quality control (30). The reads with an average base mass value lower than 20 and with a length lower than 30 bp will be removed. Quality-filtered reads were assembled using SPAdes v3.15.4 in meta mode with error correction and k-mer settings of 21, 33, and 55 (31). Scaffolds larger than 1 kb were functionally annotated using Prodigal and eggNOG-mapper v2.1.11 to characterize the functional potential of each sample (32, 33). Metagenomic binning was performed using a combination of Bowtie2 v2.3.2 (34), SAMtools v1.3.1 (35), MetaBAT2 v2.9.1 (36), MaxBin 2 v2.2.4 (37), and CONCOCT (38). Metagenome-assembled genome (MAG) quality was assessed using CheckM v1.0.7 (39), and only genomes with >90% completeness and <10% contamination were retained for subsequent analyses, guided by the Minimum Information about a Metagenome-Assembled Genome (MIMAG) standards (40). Taxonomic classification was performed using Kraken2 and Bracken (41, 42).

## RESULTS

### Mineral composition and differences in sediment analysis

**Fig 2:**
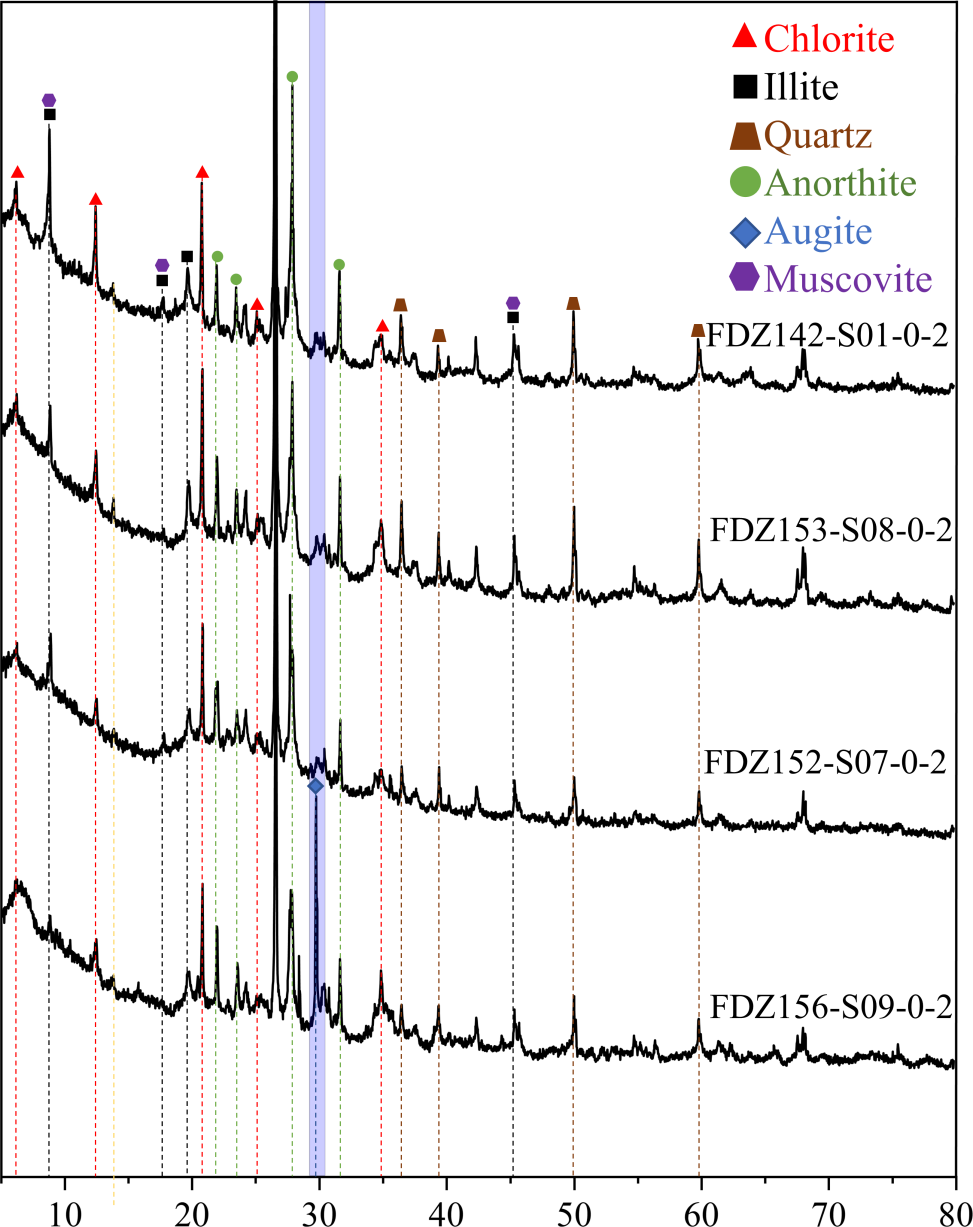
XRD results of analyzed sediments from this study. The blue column highlights the primary differences between site FDZ 156 and the other sites.

The sediments studied contain major mineral types, including anorthite, quartz, muscovite, chlorite, and illite. Notably, Station 156 shows distinctive peaks in Augite (Fig. 2). Through sampling and microscopic observations, we found a significant abundance of augite and hematite at Station 156 compared to other stations, marking a significant compositional difference.

**Fig 3:**
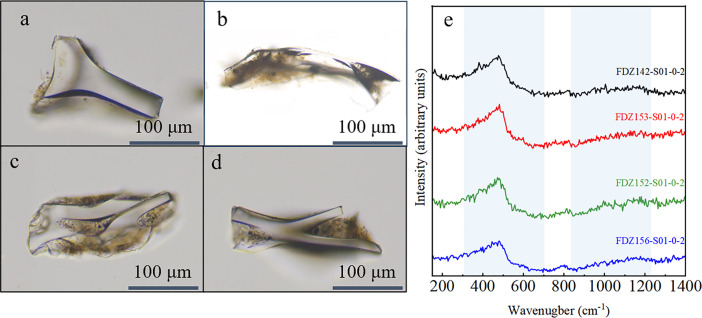
Representative glass shards from the four study sites and their Raman spectra. (**a**) FDZ-142-0-2; (**b**) FDZ-152-0-2; (**c**) FDZ-153-0-2; and (**d**) FDZ-156-0-2. (**e**) Raman spectrum showing a low-wavenumber (Lw) domain evolving from a single peak around 480–490 cm^−1^, with no shoulders near 580 cm^−1^ in most basaltic glass (45). The overall spectral profiles are highly consistent across all samples, indicating minimal compositional variation among volcanic glass at different sites. Therefore, variations in microbial communities across stations are unlikely to be driven by differences in the chemical composition of the glass itself.

The samples come from the Kermadec Trench, where the sedimentary provenance is predominantly influenced by terrigenous inputs from New Zealand (18, 43, 44). The Raman analysis of volcanic glasses from these sites, both indicative of high SiO2 content and showing a glass morphology that is clear, smooth, and less vesicular, indicates they are rhyolitic glasses (Fig. 3) (45). The Taupo Volcanic Zone (TVZ) is recognized as the world’s most active Quaternary rhyolite system (46). For example, volcanic ash from TVZ eruptions is dispersed across the North Island and the southwest Pacific by strong southwesterly winds, with traces found even in Antarctic ice cores (43, 47). Once deposited on the North Island, volcanic ash can be transported from the tectonically elevated Southern Alps (a collision zone) to the continental shelf. From there, it is carried by the Deep Western Boundary Current (DWBC) drift system and transported northward, eventually subducting into the Kermadec Trench (18).

Station FDZ156 is situated at the deepest point of the trench, in the sedimentary center, where the mineral composition suggests additional influences. Specifically, the presence of augite and hematite at FDZ156 indicates contributions from basaltic volcanic material, likely sourced from the adjacent Kermadec Ridge. For instance, basaltic andesite is reported as the primary material from Raoul and Macauley Islands within the Kermadec Ridge (48). The differences in sediment composition between stations are influenced by variations in volcanic material. The augite and hematite at FDZ156 provide a higher iron content compared to other stations. Given the role of iron in shaping microbial metabolic strategies, we hypothesized that such mineralogical differences may drive corresponding variations in microbial community composition and functional genes. This relationship is further explored in the following sections.

### Structure and composition of the microbial communities

**Fig 4:**
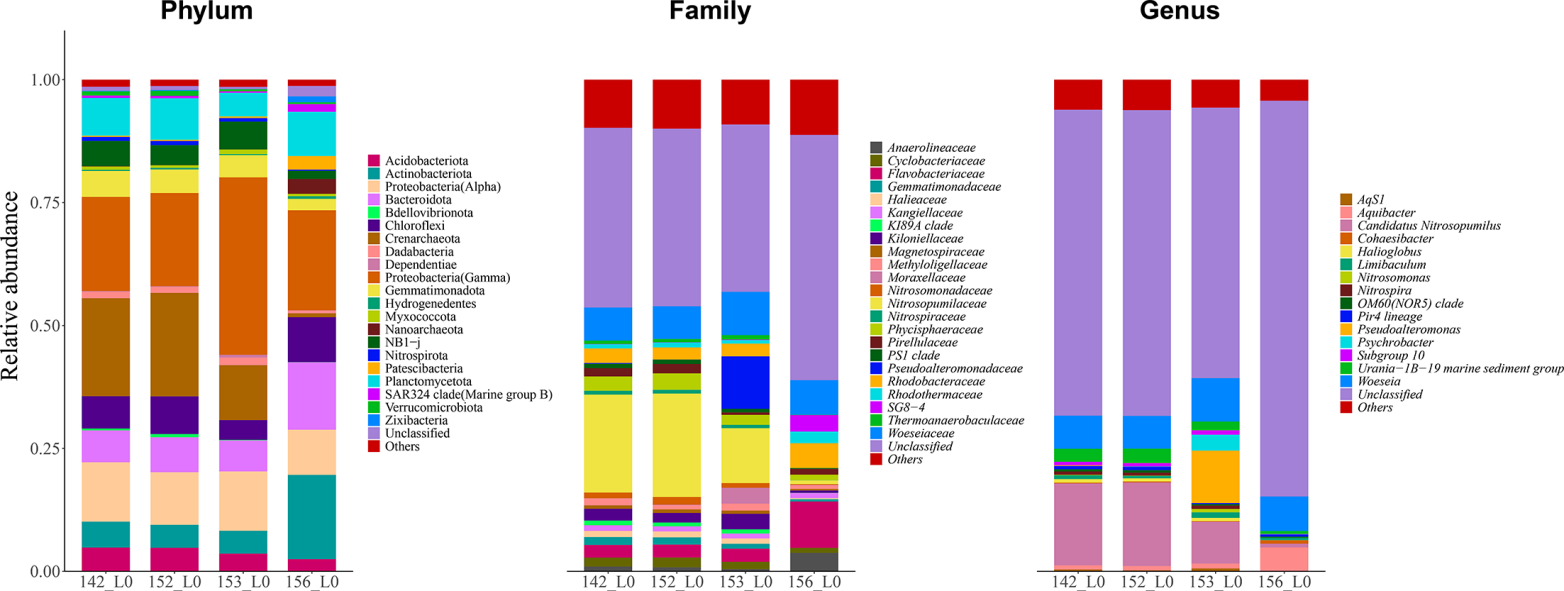
The composition of microbial communities in the four Kermadec Trench sediments at the classification level of phylum, family, and genus.

**Fig 5:**
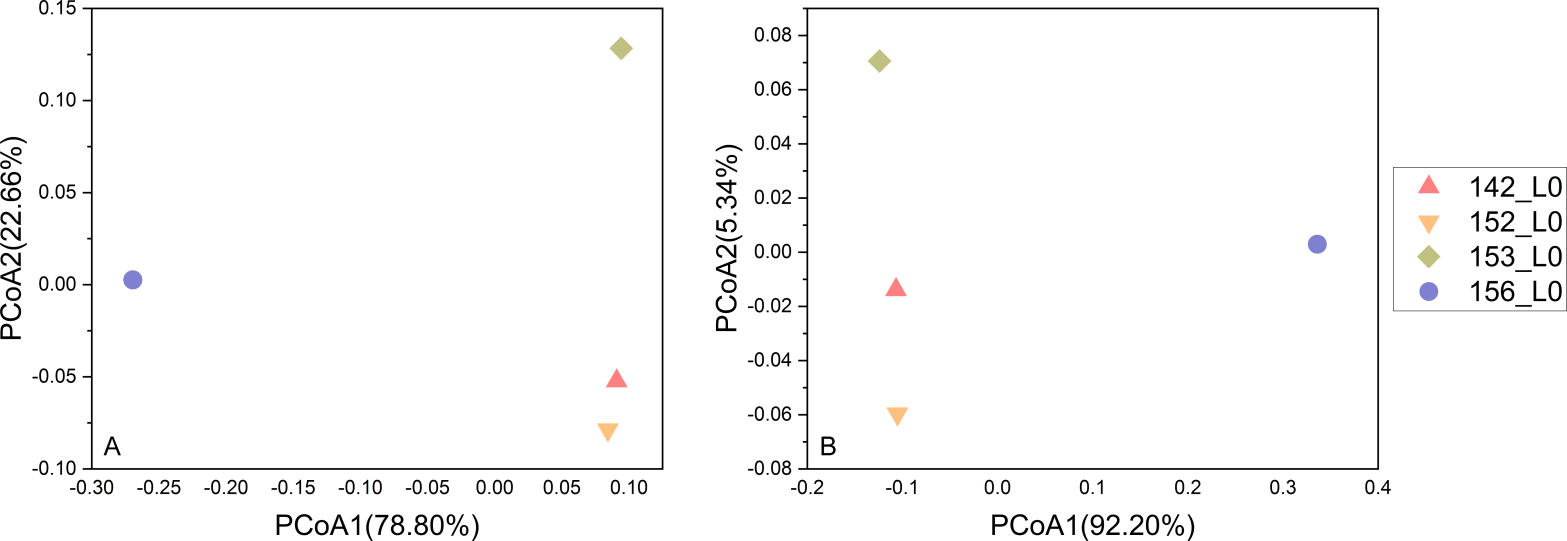
PCoA analysis of the microbial community structure in the four Kermadec Trench sediments. The results were based on the ASVs data sets. Plots in (**A**) were calculated based on weight distance, while (**B**) was calculated based on unweighted distance, respectively.

A total of 356,804 amplicon sequences were obtained from the four Kermadec Trench surface sediment samples. Each sample yielded an average of 89,201 ± 7,447 sequences. For alpha diversity analysis, read counts were normalized by rarefaction to 81,702 sequences per sample to ensure accurate comparisons of microbial diversity, composition, and structure. Shannon, Inverse Simpson, and Chao1 indices, along with observed richness, indicated lower alpha diversity in samples 153_L0 and 156_L0, with sample 156_L0 exhibiting the lowest diversity (Table 2). Microbial community composition based on 16S rRNA gene amplicon data at the phylum, family, and genus levels is presented in Fig. 4. At the phylum level, Proteobacteria (Gamma) was dominant in all samples. Crenarchaeota and Proteobacteria (Alpha) were also dominant in samples 142_L0, 152_L0, and 153_L0, while Actinobacteriota and Bacteroidota dominated sample 156_L0. At the family level, *Woeseiaceae* was dominant across all samples. *Nitrosopumilaceae* was also dominant in samples 142_L0, 152_L0, and 153_L0. Approximately half of the sequences in sample 156_L0 were unclassifiable at the family level. Among the classified sequences, *Flavobacteriaceae* and *Rhodobacteraceae* were dominant. *Pseudoalteromonadaceae* was exclusively dominant in sample 153_L0. At the genus level, a large proportion of sequences remained unclassified (62.21%, 62.18%, 55.02%, and 80.49% for samples 142_L0, 152_L0, 153_L0, and 156_L0, respectively). Within the classified fraction, *Woeseia* was the dominant genus in all samples, and *Candidatus Nitrosopumilus* was dominant in samples 142_L0, 152_L0, and 153_L0. *Urania*-1B-19 marine sediment group was also dominant in samples 142_L0 and 152_L0. *Pseudoalteromonas* was exclusively dominant in sample 153_L0 and *Aquibacter* in sample 156_L0. Microbial community structure analysis of the four abyssal surface sediment samples revealed that sample 156_L0 was distinct from the other three, while 142_L0 and 152_L0 showed greater similarity to each other (Fig. 5). These results are consistent with the alpha diversity findings. Viral metagenomic analysis revealed that most viral MAGs were unassigned, suggesting the presence of numerous novel viral species. Viral community composition at the family level is shown in Fig. S43 at https://doi.org/10.5281/zenodo.15798245. Unclassified Caudoviricetes was the most abundant family in all samples. *Autographiviridae* and other families with less than 1% relative abundance were exclusively observed in sample 156_L0, whereas *Peduoviridae* was unique to sample 153_L0. Host prediction identified *Pseudoalteromonas tetraodonis* (sample 153_L0_bin_7) as the host for *Pseudoalteromonas* phage C5a.

**TABLE 2:** The α-diversities of microbial communities from the four Kermadec Trench sediments

Index Name	Shannon	Inv_Simpson	Observed_richness	Chao
142_L0	6.0829	123.2017	2,211	2,372.6779
152_L0	6.0481	138.5978	2,080	2,215.3526
153_L0	5.8634	58.8766	2,178	2,306.4346
156_L0	5.2280	52.0501	1,303	1,414.8182

### Analysis of microbial iron-related genes

**Fig 6:**
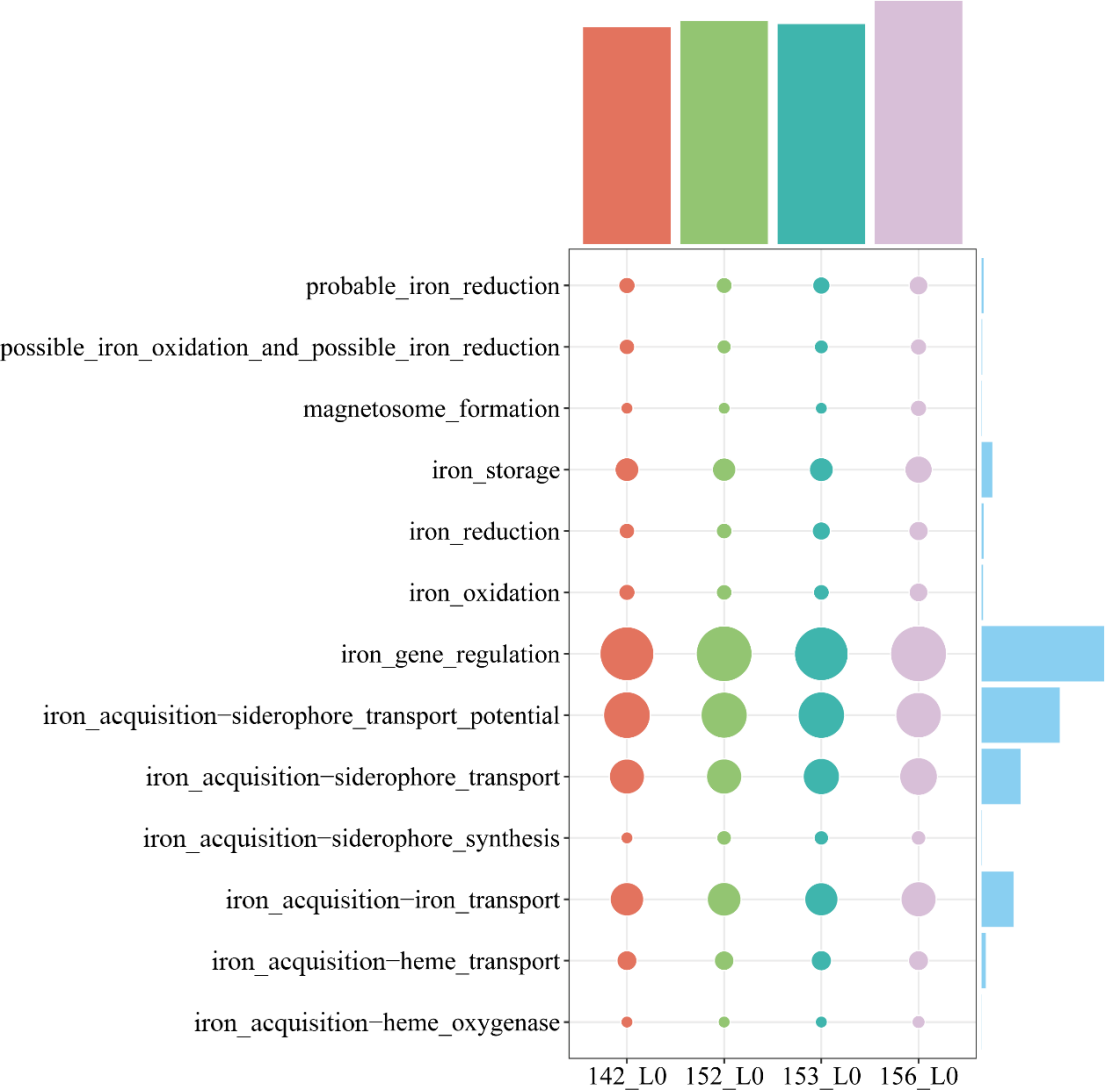
The distribution of iron-related functional genes in four Kermadec Trench sediment samples. Circle size represents the relative abundance of each gene category, normalized according to the number of protein-coding genes predicted within each metagenome. The bar charts at the top and right indicate the total normalized abundance of each sample and each gene category, respectively.

After completing assembly of the four surface sediment metagenomes, scaffolds greater than 1 kb in length from each sample were analyzed for iron-related genes, encompassing categories involved in iron acquisition (heme oxygenase, heme transport, iron transport, siderophore synthesis, siderophore transport, and siderophore transport potential), iron gene regulation, iron oxidation, iron reduction, iron storage, magnetosome formation, and putative iron oxidation and reduction (49). Subsequently, for the purpose of making the results more comparable, we normalized the output results according to the number of protein-coding genes predicted within each metagenome, and the results showed the highest abundance of microbial iron-associated genes in sample 156_L0, followed by 152_L0, with similar normalized gene counts in samples 153_L0 and 142_L0 (Fig. 6). We also attempted to identify iron-related genes in the viral sequences. However, no evidence was found to suggest direct viral involvement in iron metabolism.

**Fig 7:**
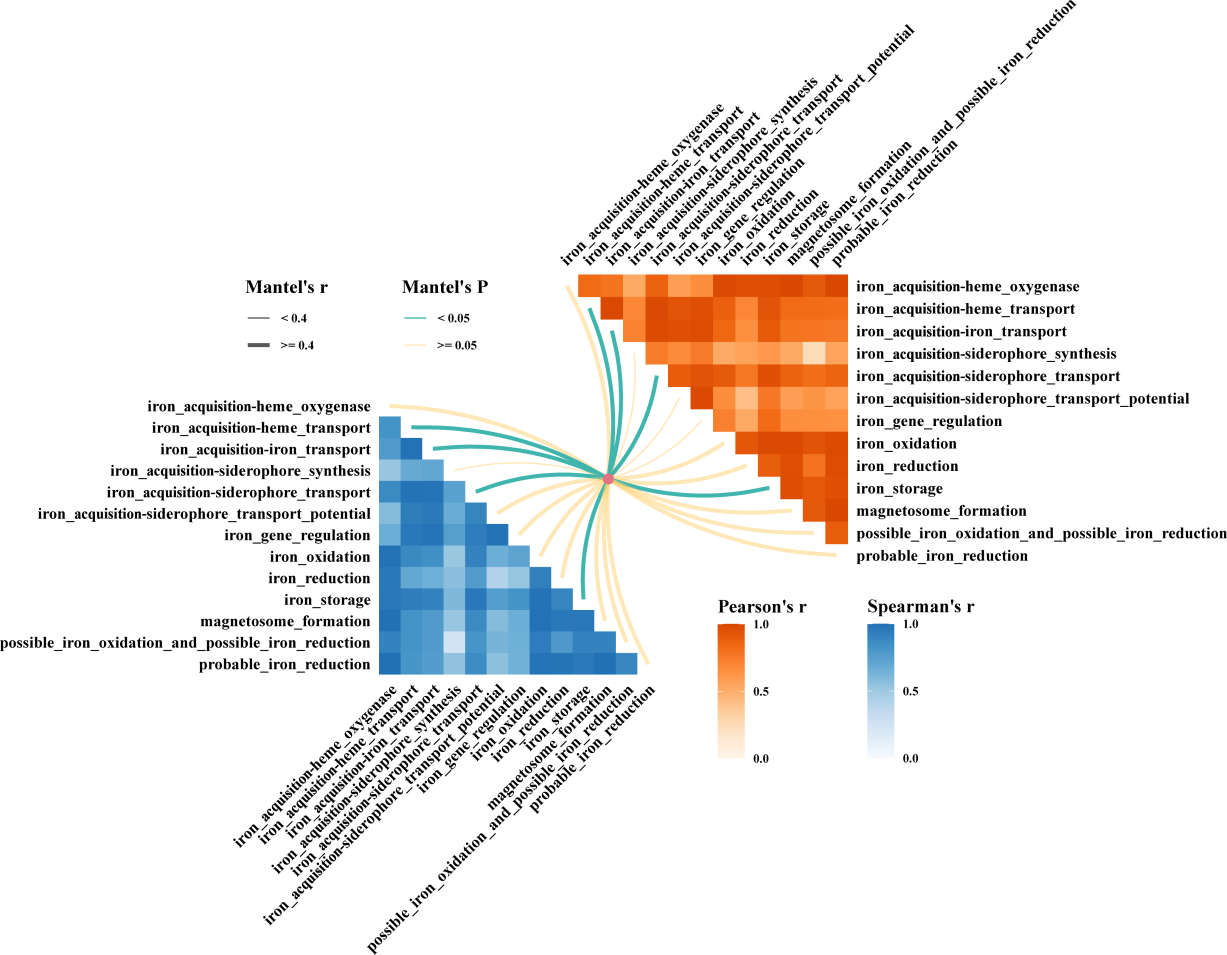
Mantel analysis of the relationship between microbial communities and iron functional genes in four Kermadec Trench sediments. Line thickness indicates Mantel’s *r* values, and line color represents statistical significance. The blue part was based on the Spearman correlation coefficient, and the red part was based on the Pearson correlation coefficient.

We acquired information on the microbial communities in the four volcanic ash-affected surface sediments of the Kermadec Trench, but it was not clear to us which categories of iron-associated genes were mainly influencing the structure of these microbial communities. To address this, we employed correlation analyses based on 16S rRNA gene amplicon data to investigate the relationship between microbial community structure and various iron-related gene categories. The results, based on Pearson and Spearman correlation coefficients, indicated that microbial community structure in these volcanic ash-affected sediments was primarily influenced by four iron-related gene categories: iron acquisition–heme transport, iron acquisition–iron transport, iron acquisition–siderophore transport, and iron storage (Fig. 7).

The abundance of unclassifiable microorganisms within the microbial communities of these samples suggested a high diversity of novel and undiscovered microbial taxa in the volcanic ash-affected abyssal surface sediments. To investigate the potential role of these sediment microbes in abyssal iron cycling and to determine their taxonomic affiliations, we retrieved a large number of metagenome-assembled genomes (MAGs) from the metagenomic data. To ensure high-quality results, we retained only MAGs with genome completeness exceeding 90% and contamination below 10% for subsequent analyses.

**Fig 8:**
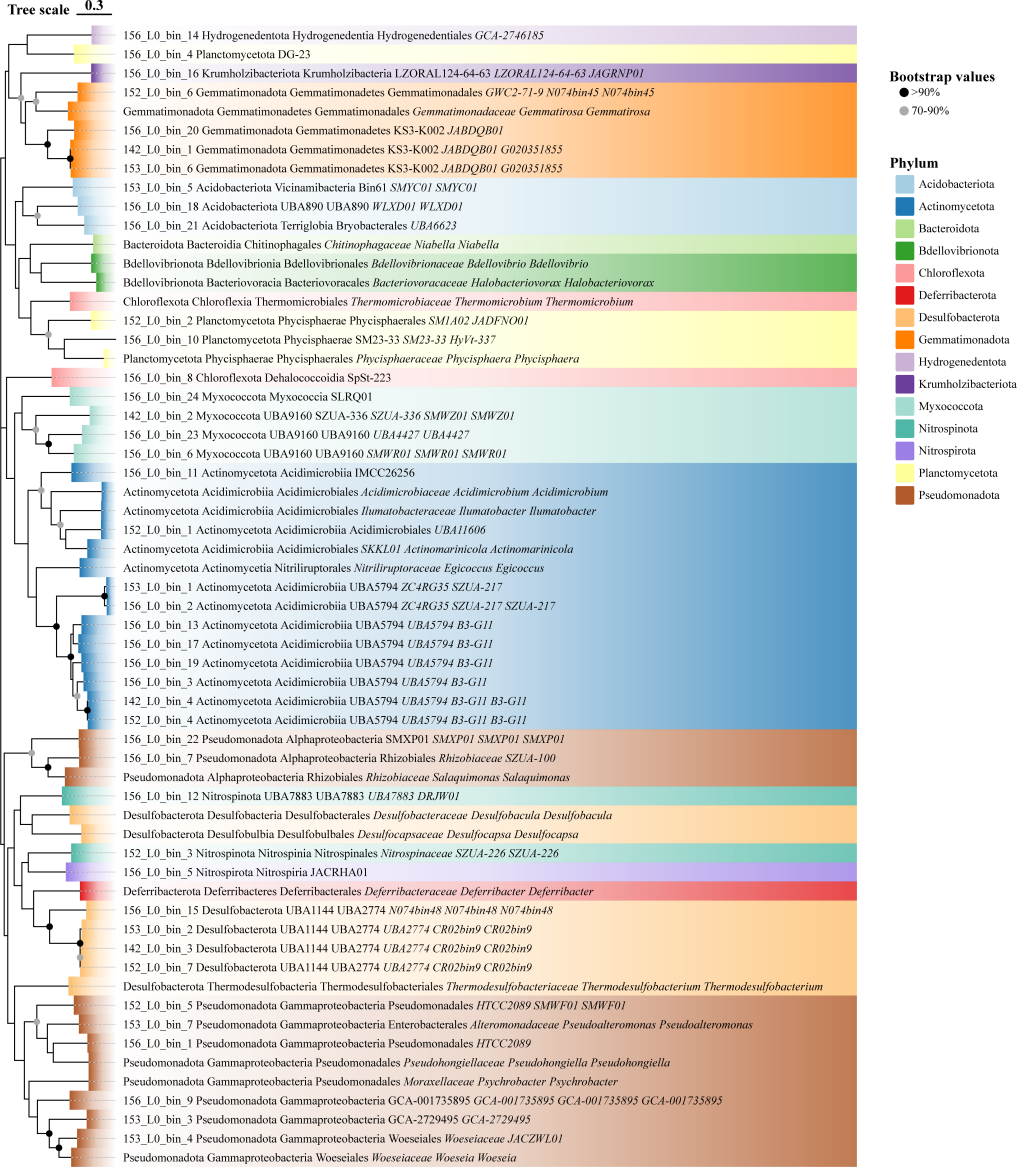
Phylogenetic trees constructed from refined MAGs and the most relevant reference genomes jointly provided by Kraken2 and Bracken from four Kermadec Trench sediment metagenomic data.

**Fig 9:**
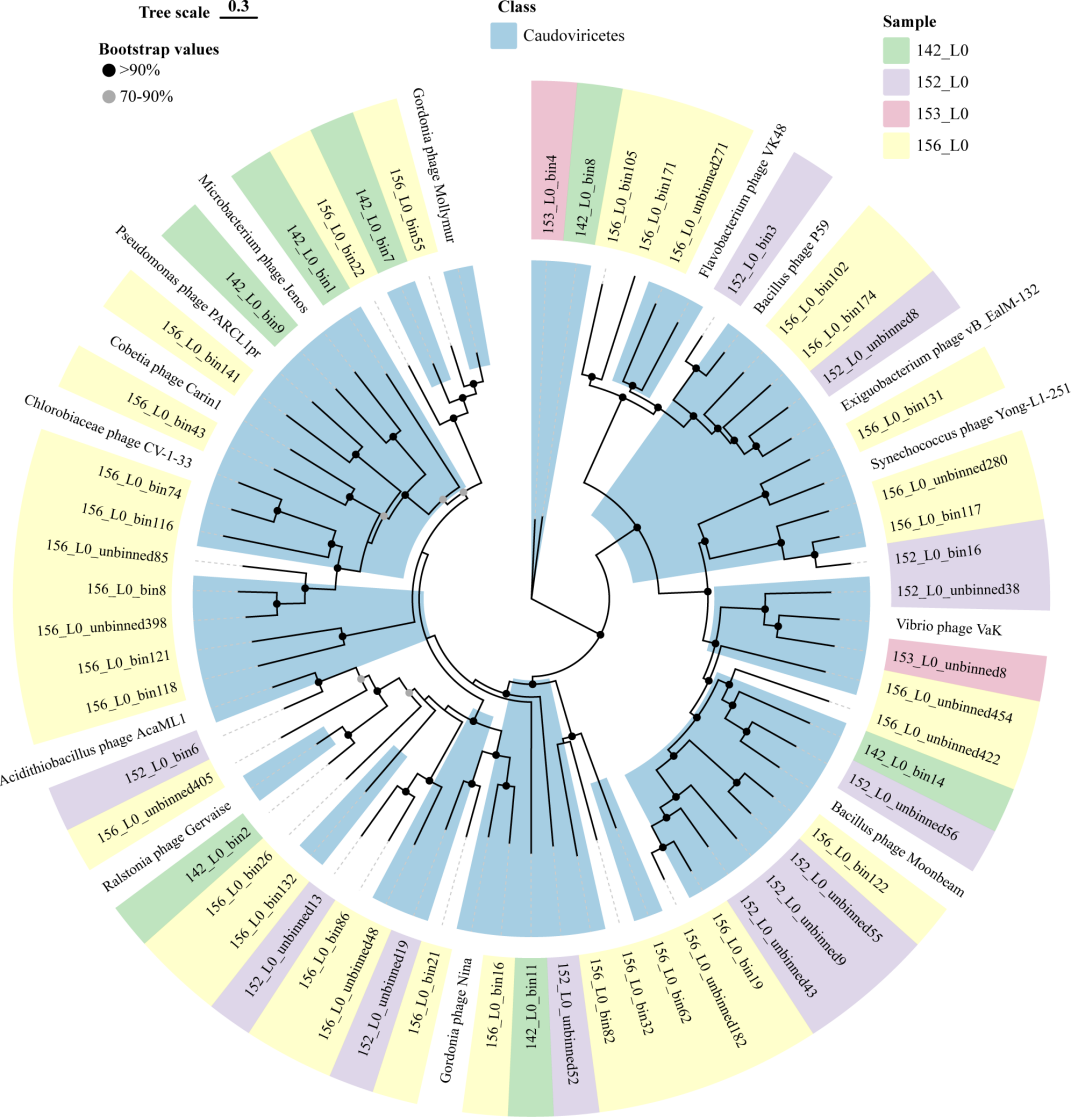
Phylogenetic tree constructed by high-quality viral MAGs obtained from metagenomic data from four Kermadec Trench sediments. We obtained more accurate taxonomic information and phylogenetic analysis for the high-quality viral MAGs, which were generated using the ViWrap (53), which integrates CheckV for quality estimation. The taxonomic classification was performed with PhaBOX (54), and the phylogenetic relationships were visualized using IQ-TREE (55), with ModelFinder Plus (MFP) used for model selection and ultrafast bootstrap analysis (1,000 replicates) to assess branch support. Tree visualization was done using tvBOT (56). However, the majority of these MAGs could not be annotated using existing databases, suggesting that they may belong to previously unidentified viral taxa.

Species annotation of each selected MAG was performed using Kraken2 and Bracken. Based on the annotated species information, relevant reference genome sequences were downloaded, and phylogenetic analyses were conducted using GTDB-Tk (50, 51), which constructs trees based on bacterial single-copy marker genes, to compare each MAG sequence with its annotated reference genomes. Additionally, average nucleotide identity (ANI) was calculated using ANI calculator (52) to compare MAG genome sequences with those of phylogenetically related microbial species. Results indicated that all MAGs with >90% completeness and <10% contamination represented novel bacterial taxa, with the highest ANI reaching only 70.13%. Phylogenetic trees and ANI comparison results are provided in Fig. S1 to S42 at https://doi.org/10.5281/zenodo.15798245. To comprehensively present the taxonomic and phylogenetic information of all refined MAGs, these MAG genomes and related reference genomes were analyzed together using GTDB-Tk (Fig. 8). Similarly, accurate taxonomic and phylogenetic information was obtained for high-quality viral MAGs generated using ViWrap (53). Taxonomic classification was performed using PhaBOX (54), and phylogenetic relationships were visualized using IQ-TREE (55) and tvBOT (56). However, most viral MAGs remained unannotated using existing databases, suggesting they belong to previously unidentified viral taxa (see Fig. S44 at https://doi.org/10.5281/zenodo.15798245 and Fig. 9). Furthermore, we examined whether these MAGs harbored iron-related genes, indicating their potential involvement in abyssal iron cycling. All selected MAGs were found to carry iron-related genes (see Table S1 at https://doi.org/10.5281/zenodo.15798245). However, the number of iron-related genes within these MAGs was significantly lower than the total number of iron-related genes identified in the four metagenomes (see Table S2 at https://doi.org/10.5281/zenodo.15798245), suggesting that a substantial number of iron-related genes are distributed across other microbial genomes.

## DISCUSSION

Microbial communities inhabiting hadal trenches exhibit distinct characteristics shaped by extreme environmental conditions. Notably, microbial activity has been observed to be relatively elevated in the deepest sections of certain hadal trenches (57). However, chemolithotrophic populations in hadal waters are less abundant than in upper waters, and species-level niche partitioning has been identified among dominant taxa within these communities (58). This disparity underscores the high specialization of microbial communities in trench sediments, warranting further investigation. Benthic, microbially mediated processes play a significant role in global elemental cycling (59). Iron cycling, in particular, is crucial within biogeochemical cycling, influencing diverse environmental processes. A wide range of microorganisms utilize iron redox transformations for energy, and our understanding of their physiological characteristics, ecological functions, and environmental significance is rapidly advancing (60). Volcanic ash, a significant component of marine sediments, is enriched in iron and manganese (61). Prior research indicates that terrestrial volcanism is a substantial source of these micronutrients for the global ocean (62, 63). With advancements in deep-sea engineering technology, the study of microorganisms in hadal trench sediments is increasingly feasible. In this study, we investigated the relationship between volcanic ash sediments and microbial communities, revealing a strong association between iron and community formation and ecosystem evolution.

Our study revealed elevated concentrations of augite and hematite in sample 156_L0, indicating distinctive depositional environments potentially influenced by geological conditions and volcanic inputs (18). The mineral accumulation observed at site 156 is likely attributed to basaltic volcanic material originating from the nearby Kermadec Ridge (48). Given the probable role of augite and hematite in providing iron, iron-related taxa may be selectively enriched. These findings suggest that mineral composition may play a critical role in shaping microbial community structure and regulating ecological functions, particularly through iron cycling.

The elevated iron content in sample 156_L0, coupled with its lowest microbial diversity, suggests a potential influence or correlation between iron and microbial community structure. At the phylum level, Gammaproteobacteria dominated in all samples. This bacterial phylum is abundant in deep-sea environments, playing a critical role in iron oxidation processes (64–66). These microbially mediated or catalyzed processes can be utilized for cellular functions (66). Furthermore, Gammaproteobacteria are sensitive to iron addition during methane oxidation (67). *Woeseiaceae*, a family within Gammaproteobacteria and the dominant family in all samples, likely facilitates metal-sulfide dissolution through extracellular electron transfer (68). Notably, the dominant genus *Woeseia* within this family exhibits a positive correlation with higher iron concentrations and is highly specific to ferromanganese nodules, indicating its potential role in nodule formation (69–71). Actinobacteriota, a dominant in sample 156_L0, are common iron-reducing bacteria in soils and sediments (72). Furthermore, they play a significant role in iron acquisition and transport, producing various siderophores to capture iron for growth and metabolism (73). Zero-valent iron has been shown to increase the abundance of Bacteroidota, also a dominant in sample 156_L0 (74). Additionally, *Bacteroides* sp. W7 can reduce ferric iron and transfer electrons to ferric iron during growth using carbon compounds (75). *Flavobacterium psychrophilum*, within the *Flavobacteriaceae* family of Bacteroidota, can acquire iron from transferrin and hemoglobin (76). *Flavobacterium columnare* produces siderophores that specifically bind ferric iron, facilitating its cellular transport (77). Furthermore, Alphaproteobacteria have been reported to possess multiple iron-responsive genes (78). Crenarchaeota has been suggested as a dominant phylum in low-density ferromanganese deposit samples (79). The distinct microbial community structure of sample 156_L0 may be attributed to ecological distance resulting from mineral composition discrepancies.

Iron is essential for nearly all organisms, playing a pivotal role in numerous metabolic processes. During microbial iron-related gene analysis, we observed that sample 156_L0 exhibited the highest abundance of microbe-mediated iron-associated genes. Concurrently, sample 156_L0 displayed a higher concentration of hematite, potentially providing a more favorable environment for iron-interacting microbes. In the study of viruses, we analyzed the high-quality viral MAGs we obtained for iron-related genes, and the result was that these viruses did not directly carry relevant iron metabolism-related genes. However, in our host analysis of these viruses, we found that some of the viruses’ hosts were able to directly participate in iron metabolism and iron cycling in their corresponding environments. For instance, *Pseudoalteromonas* phage C5a is hosted by the genus *Pseudoalteromonas*, and our microbial community composition analysis confirmed the relatively high abundance of *Pseudoalteromonas* as a dominant microorganism in the Kermadec abyssal surface sediments. This observation supports previous studies highlighting that *Pseudoalteromonas* is notably abundant in cold environments and frequently associated with deep-sea sediment habitats (80). Notably, *Pseudoalteromonas* contributes to iron metabolism by producing unique siderophores, such as lystabactins, which facilitate iron acquisition and utilization (81).

The distinct abundance of iron-related genes in sample 156_L0 suggests a strong adaptation of its microbial community to the high-iron environment. The structure of microbial communities is closely linked to four gene categories: iron acquisition–heme transport, iron acquisition–iron transport, iron acquisition–siderophore transport, and iron storage. In particular, the ABC-type (ATP-binding cassette) transporters, which are integral to major iron uptake systems, were generally more abundant in 156_L0 (82). Complementing this, the Ton system (TonB/ExbB/ExbD inner membrane complex), which may play a pivotal role in mediating iron acquisition under high-iron conditions, also showed elevated abundance in 156_L0 (83). Several well-known genes related to iron transport systems, such as *Hmu*, *Fbp*, *Yfe*, and *Pir*, were particularly enriched at this site. Together, these two transport systems appear to work synergistically, equipping the 156_L0 microbial community with robust strategies to thrive in iron-enriched conditions (84).

Similarly, iron storage genes, such as those encoding ferritin, were more abundant in 156_L0. Ferritins represent a specialized family of proteins that serve as iron storage containers, facilitating the incorporation of intracellular iron in a bioavailable and nontoxic form (85). This combination of abundant iron acquisition, transport, and storage genes highlights the adaptive advantage of the microbial community in 156_L0 under iron-rich conditions (85). The observed differences in gene abundance across samples align with the hypothesis that environmental iron availability significantly influences microbial community composition. High iron concentrations in 156_L0 may have selected for taxa with enhanced iron acquisition and storage capabilities, thereby driving community structure differences.

The phylogenetic tree and ANI indicate that a significant number of novel bacterial taxa are likely to be present in trench sediments. It is proposed that several novel bacterial taxa may be associated with iron metabolism, suggesting the potential for their future isolation and characterization. We also found that genes associated with the iron acquisition–siderophore transport potential category were generally present in most of the MAGs across the four samples. However, all MAGs classified as Actinomycetota lacked these genes, while those with iron acquisition-siderophore transport genes likely utilize known transport systems for iron acquisition (73). In MAGs classified under the new phylum Myxococcota, the presence of iron-related gene categories was consistent with the proportions observed across all samples. It has been reported that Myxococcota species have the capacity to transport ferrous iron, suggesting that they may utilize broad and conserved iron metabolic pathways, a strategy that could be widely applicable to adapting to deep-sea volcanic ash sediment habitats (86). Additionally, genes responsible for magnetosome biomineralization and magnetotaxis were identified in Myxococcota, which are unique organelles in magnetotactic bacteria. Magnetosomes consist of membrane-bound, nano-sized magnetite (Fe₃O₄) and/or greigite (Fe₃S₄) crystals (87). Understanding the role of these bacteria in iron metabolism could provide insights into biogeochemical cycles in deep-sea ecosystems, particularly how the iron from volcanic ash input influences ecosystem evolution and nutrient cycling.

### Conclusion

Our study elucidated the impact of volcanic ash on microbial communities in deep-sea sediments, specifically within the Kermadec Trench. We found that differences in iron concentrations across sediment samples led to notable variations in microbial diversity, with the highest diversity seen in samples with lower iron content. This suggests that iron plays a key role in shaping microbial community structure in these environments. In iron-rich sediments, dominant microbial groups, such as Actinobacteriota, were more prevalent, suggesting that iron influences microbial community composition. Iron-related functional gene analysis highlighted the importance of iron acquisition, transport, and storage in determining microbial community structure. These findings offer valuable insights into how volcanic ash deposits influence microbial ecosystems and how these microbes contribute to deep-sea biogeochemical cycles, particularly iron cycling. In particular, our results underscore the adaptive strategies employed by microbial communities in response to localized iron enrichment, such as the enrichment of ABC-type transporters and the Ton system. This highlights the broader ecological significance of mineral-driven selection pressures in abyssal ecosystems. To deepen our understanding of these processes, future studies might explore *in situ* approaches combined with methods such as metatranscriptomics and isotope probing that could help uncover how microbial metabolic processes respond to iron enrichment in deep-sea environments. Laboratory-based simulations that replicate the geochemical changes triggered by volcanic ash deposition could also offer valuable insights into how microbial communities respond to such environmental disturbances. Comparative studies across different trench systems and ash deposition types would also help generalize the ecological roles of volcanic ash in shaping deep-sea biogeochemical processes.

## Data Availability

Raw sequencing reads for all samples were deposited in the NCBI database (http://www.ncbi.nlm.nih.gov/) under BioProject accession number: PRJNA1222810 for the microbial amplicon data sets and PRJNA1216632 for the microbial metagenomic data sets of all the surface sediments in this study.
